# Circulating rotavirus strains in children with acute gastroenteritis in Iran, 1986 to 2023 and their genetic/antigenic divergence compared to approved vaccines strains (Rotarix, RotaTeq, ROTAVAC, ROTASIIL) before mass vaccination: Clues for vaccination policy makers

**DOI:** 10.1016/j.virusres.2024.199411

**Published:** 2024-06-03

**Authors:** Somayeh Jalilvand, Tayebeh Latifi, Atefeh Kachooei, Mahtab Mirhoseinian, Somayeh-Sadat Hoseini-Fakhr, Farzane Behnezhad, Farzin Roohvand, Zabihollah Shoja

**Affiliations:** a Department of Virology, School of Public Health, Tehran University of Medical Sciences, Tehran, Iran; b Department of Virology, School of Medicine, Iran University of Medical Sciences, Tehran, Iran; c Department of Virology, Pasteur Institute of Iran, Tehran, Iran; d Research Center for Emerging and Reemerging Infectious Diseases, Pasteur Institute of Iran, Tehran, Iran

**Keywords:** Rotavirus, VP7, VP4, Genotype, lineage, Vaccine

## Abstract

In the present study, first, rotaviruses that caused acute gastroenteritis in children under five years of age during the time before the vaccine was introduced in Iran (1986 to 2023) are reviewed. Subsequently, the antigenic epitopes of the VP7 and VP4/VP8 proteins in circulating rotavirus strains in Iran and that of the vaccine strains were compared and their genetic differences in histo-blood group antigens (HBGAs) and the potential impact on rotavirus infection susceptibility and vaccine efficacy were discussed. Overall data indicate that rotavirus was estimated in about 38.1 % of samples tested. The most common genotypes or combinations were G1 and P[8], or G1P[8]. From 2015 to 2023, there was a decline in the prevalence of G1P[8], with intermittent peaks of genotypes G3P[8] and G9P[8]. The analyses suggested that the monovalent Rotarix vaccine or monovalent vaccines containing the G1P[8] component might be proper in areas with a similar rotavirus genotype pattern and genetic background as the Iranian population where the G1P[8] strain is the most predominant and has the ability to bind to HBGA secretors. While the same concept can be applied to RotaTeq and RotasIIL vaccines, their complex vaccine technology, which involves reassortment, makes them less of a priority. The ROTASIIL vaccine, despite not having the VP4 arm (P[5]) as a suitable protection option, has previously shown the ability to neutralize not only G9-lineage I strains but also other G9-lineages at high titers. Thus, vaccination with the ROTASIIL vaccine may be more effective in Iran compared to RotaTeq. However, considering the rotavirus genotypic pattern, ROTAVAC might not be a good choice for Iran. Overall, the findings of this study provide valuable insights into the prevalence of rotavirus strains and the potential effectiveness of different vaccines in the Iranian and similar populations.

## Highlight

• Overall, the most common genotypes or combinations were G1 and P[8], or G1P[8].
• From 2015 to 2023, intermittent peaks of genotypes G3P[8] and G9P[8] were detected.
• Rotarix vaccine might be proper in Iran.
• The same concept of Rotarix can be applied to RotaTeq and RotasIIL vaccines in Iran.
• ROTAVAC may not provide as much protection as the other vaccines in Iran.

## 1 Introduction

Prior to the introduction of rotavirus vaccine, rotavirus was the primary cause of acute gastroenteritis in children under five years of age worldwide, with high mortality rates particularly in low- and middle-income countries (LMICs) (Tate et al., 2016). In 2006, two types of human live- attenuated rotavirus vaccines became available: Rotarix (GlaxoSmithKline Biologicals, Belgium), which contained the attenuated G1P[8] strain 89–12, and RotaTeq (Merck & Co, Inc,) a pentavalent human-bovine mono-reassortant vaccine containing G1 (WI79 strain), G2 (SC2 strain), G3 (WI78 strain), G4 (BrB strain), P7[5] (WC3 strain) and P1A[8] (WI79 strain) (Ruiz-Palacios et al., 2006; Vesikari et al., 2007, 2006). These vaccines were subsequently incorporated into national immunization programs by the World Health Organization (WHO), primarily in LMICs (WHO, 2009). Additionally, two other human live- attenuated rotavirus vaccines, ROTAVAC (a monovalent human-bovine [116E] rotavirus vaccine; Bharat Biotech) and ROTASIIL (a live attenuated human-bovine reassortant rotavirus pentavalent vaccine, covering serotypes G1, G2, G3, G4, and G9; Serum Institute of India) have been WHO‐prequalified and have demonstrated efficacy similar to that of RotaTeq and Rotarix in India and several LMICs (Bhandari et al., 2014; Chilengi et al., 2021; Isanaka et al., 2021; Kulkarni et al., 2017).

Rotavirus, belongs to the *Sedoreoviridae* family and is a non-enveloped, double-stranded (DS) RNA virus. It contains 11 segments that encode six structural (VP1-VP4, VP6, VP7) and five or six nonstructural proteins (NSP1‐NSP5/6). Based on the serological cross-reactivity to the VP6 protein and molecular characterization of VP6 gene, 10 rotavirus groups (A-J) are described (Banyai et al., 2017; Matthijnssens et al., 2012; Mihalov-Kovacs et al., 2015). Furthermore, two new tentative groups, rotavirus K and L, are identified based on their genome sequence identities (Johne et al., 2019, 2023). But only groups A, B, C, and H infect humans, among which group A rotavirus is the most medically important group (Doro et al., 2015). Throughout this review "rotavirus" is used to indicate “group A rotavirus”. The glycoprotein VP7 and protease-sensitive protein VP4 are used to classify rotaviruses into G and P types, (binary classification), with a total of 42 G and 58P genotypes recognized globally (http://rega.kuleuven.be/cev/viralmetagenomics/virus-classification/rcwg, updated 25 Mar, 2021). Additionally, classification based on all 11 genomic RNA segments has been documented for rotaviruses where Gx‐P[x]‐Ix‐Rx‐Cx‐Mx‐Ax‐Nx‐Tx‐Ex‐Hx define the genotype of the VP7‐VP4‐VP6‐VP1‐VP2‐VP3‐NSP1‐NSP2‐NSP3‐NSP4‐NSP5/6 genes, respectively (Matthijnssens et al., 2008a, 2008b). The most common rotavirus strains associated with human gastroenteritis are G1, G2, G3, G4, G9, and G12 types, combined with either P[8], P[4] or P[6] types (Banyai et al., 2012; Doro et al., 2014; Gentsch et al., 2005; Santos and Hoshino, 2005). In this context, G1P[8], G2P[4], G3P[8], G4P[8], G9P[8], and G12P[8] strains are responsible for 90 % of rotavirus related- acute gastroenteritis cases, with varying geographical distribution (Banyai et al., 2012; Doro et al., 2014; Gentsch et al., 2005; Santos and Hoshino, 2005). Of note, the G1P[8] strain continues to have a high prevalence in most regions while zoonotic genotypes such as G5 and P[11] (porcine) and G6, G8 and P[6] (bovine) are common in developing countries where they cause infections in humans (Iturriza Gómara et al., 2004; Malasao et al., 2018; Matthijnssens et al., 2008a; Todd et al., 2010).

Since global rotavirus vaccination, rotavirus-caused mortality rates are highly declined. In this context, results of a cross-sectional study on Global Burden of Disease (GBD) indicated that use of rotavirus vaccines prevented over 28,000 deaths (Troeger et al., 2018). Similarly, results of another more recent study indicated that administration of rotavirus vaccines prevented around 139,000 children deaths (corresponding to around 37 % of rotavirus-related deaths) between 2006 and 2019 (Clark et al., 2023). Several other studies reported the impact of rotavirus-vaccination in declining the proportion of children hospitalized with rotavirus-infection (Aliabadi et al., 2019; Burnett et al., 2020). Based on the analyses of a systematic review in 2019 alone, an estimated 1.76 million hospitalizations were attributed to rotavirus infection while, the introduction and coverage of rotavirus vaccines have played a crucial role in preventing over 500,000 hospitalizations in the same year. Indeed, findings of the Global Rotavirus Surveillance Network (GRSN) indicated that from 2008 to 2016 the detection rate for rotavirus infection in countries without a national immunization program was around 38 % of the children with acute gastroenteritis whereas in those that introduced the vaccine, the detection rate was around 23.0 % (Aliabadi et al., 2019). Similarly, based on the published studies from 2006 to 2019 (Burnett et al., 2020), a median reduction of 59 % in rotavirus hospitalizations after rotavirus vaccine introduction was reported. Interestingly, results of these studies indicated higher reduction of hospitalizations for high-income countries (HICs) compared to LMICs which might be due to the differences in vaccine efficacy, effectiveness, and coverage between these countries. However, in general rotavirus-vaccination is considered as the most effective strategy to prevent rotavirus-caused deaths and hospitalizations of the children worldwide.

In Iran, based on a proportion modeling of rotavirus cases in 2016, it was estimated that rotavirus-infection caused approximately 156 deaths with a mortality rate of 1.9 (per 100,000; 95 % UI, 0.7–4.5) among children under five years of age (Troeger et al., 2018). Additionally, the average rate of rotavirus detection was found to be around 40 % in children under five years old who were hospitalized due to acute gastroenteritis (Jalilvand et al., 2018). These findings highlighted the importance and necessity of implementing rotavirus vaccination programs in Iran to reduce the associated morbidity and mortality rates. However, before introducing a rotavirus vaccine, it is essential to analyze parameters such as prevalence, circulating genotypes (G/P) patterns and lineages, and compare them with the genotypes, lineages, and antigenic epitopes of available vaccines (Rotarix, RotaTeq, ROTAVAC, and ROTASIIL). Such analyses could be based on the related reported data (which are available from 1986 to 2023) to update our understanding of the situation and assess the pre-vaccine scenario. It is worth mentioning that the VP7 and VP4/VP8 proteins are considered the primary targets for creating protection through vaccination. The VP7 glycoprotein is a major neutralization antigen of rotavirus, and antibodies against this protein neutralize the virus by inhibiting its decapsidation (Ludert et al., 2002). On the other hand, the VP4 protein is a nonglycosylated protein that is cleaved (by proteolysis) into two subunits, VP8* and VP5*, and enhances viral infectivity (Espejo et al., 1981). According to the phylogenetic analysis of the VP7 sequence of various rotavirus genotypes, including G1-G4, G9, and G12, from the global rotavirus database, these genotypes are clustered into several separate evolutionary lineages. In this context, G1 (Phan et al., 2007a), G2 (Dong et al., 2016; Trinh et al., 2010), G3 (Degiuseppe et al., 2014; Gómez et al., 2014), G4 (Ianiro et al., 2015), G9 (Phan et al., 2007c) and G12 (Wangchuk et al., 2014) have lineages of I-XI, I-VI, I-IV, I-VII, I-VI and I-IV, respectively. The VP7 trimer consists of two antigenic epitopes, 7–1 and 7–2, with 7–1 further divided into 7–1a and 7–1b (Aoki et al., 2009). Similarly, the phylogenetic analysis of the VP4/VP8 sequence of P[8], P[4], and P[6] genotypes from the global rotavirus database reveals separate evolutionary lineages. Accordingly, P[8] (Phan et al., 2007b), P[4] (Espínola et al., 2008) and P[6] (Bányai et al., 2004; Martella et al., 2006) have lineages of I-IV, I-V, and I-V, respectively. Structural studies have shown that VP8* forms a globular head on top of a stalk shaped by VP5. The VP8* head contains four surface-exposed antigenic epitopes (8–1 to 8–4) that are expected to contain 25 amino acids (Zeller et al., 2012). The strains/lineages found in the available vaccines may differ from the circulating rotavirus genotypes in various countries. Additionally, different lineages within the same genotype may have distinct antigenic properties due to amino acid changes in antigenic regions, which could potentially allow the virus to evade the immune response. Furthermore, the ABH and Lewis histo-blood group antigen family (HBGA), which serve as rotavirus receptors, are important factors to consider when studying the potential genetic sources of variation in oral vaccine outcomes (Hu et al., 2012; Huang et al., 2012; Liu et al., 2012). The HBGAs, which are ABH and Lewis glycans, can be found on the surface of epithelial cells and in mucosal secretions. The expression of HBGA can vary genetically, which can impact the susceptibility to infection by different rotavirus strains and the effectiveness of rotavirus vaccination (Nordgren et al., 2014). Therefore, when planning the launch of rotavirus vaccination, it is important to consider the differences between the strains/lineages and genetic/antigenic background in the vaccines and the circulating rotavirus strains in the targeted region/country. Indeed, continuous monitoring of rotavirus genotypes prior to implementing a national rotavirus vaccination program provides clues for the vaccination policy makers to select the best vaccine based on the predominant strains in the region. Additionally, this continuous monitoring can also identify the potential emergence of uncommon or novel types in the community, which may pose challenges to the effectiveness of the available vaccines.

The present study reviews the prevalence and profile changes of the rotavirus strains reported from Iran before introduction of rotavirus vaccine into the national immunization program (From 1986 to 2023) with a particular emphasis on the distribution patterns of circulating genotypes/sublineages as well as genetic/antigenic and structural differences of specially VP7 and VP8 antigens compared to that of the rotavirus vaccine strains (Rotarix, RotaTeq, ROTAVAC, and ROTASIIL). The information provided in this review might assist the vaccination policy makers to select the best rotavirus vaccine for national/regional vaccination programs.

## 2 Material and methods

### 2.1 Data collection

A total of 41 relevant studies (Abdolvahhab Moradi, 2001; Ataei Pirkooh and Shahrabadi, 2007; Azaran et al., 2016, 2018; Eesteghamati et al., 2009; EMAM et al., 2008; Farahtaj et al., 2007; Ghorashi et al., 2011; Hamkar et al., 2010; Jadali et al., 2012; Kachooei et al., 2023; Kargar and Akbarizadeh, 2012; Kargar et al., 2012a, 2014, 2011; Kargar et al., 2012b; Kazemi et al., 2006; Ketabi et al., 2023; Khalili et al., 2004; Lorestani et al., 2019; MODARES et al., 2008; MODARRES et al., 2011; Mohammad Kargar, 2007; Motamedi-Rad et al., 2020; Motamedifar et al., 2013; Mozhgani et al., 2012; Najafi et al., 2013; Nasab et al., 2016; Sadeghian et al., 2010; Safieh Amini, 1990; Samarbafzadeh et al., 2005; Shahrazad Modarres, 1995; Shams et al., 2020; Sharifi-Rad et al., 2015; Shokrollahi et al., 2014; Zarnani et al., 2004) were identified in Iran through searches of Medline, Current Contents, PubMed, and references from relevant articles. The search utilized key words such as "prevalence," "molecular epidemiology," "rotavirus," "genotype," and "Iran." These studies focused on evaluating the positive rate and circulating strains of rotavirus in children under five years of age. The data from these studies, covering the period from 1986 to 2023, were reviewed. Furthermore, nucleotide sequences of rotavirus genotypes VP7 G1-G4, G8, G9, and G12, as well as VP4/VP8 P[8], P[4], P[6], and P[9] in Iran, were obtained from the NCBI-published database. Reference rotavirus isolates representing various genotypes and sub-genotypic lineages were also included, while rotavirus genotypes with partial and/or single sequences were excluded from the analysis.

### 2.2 Genetic, phylogenetic and structural analysis

The nucleotide sequences of rotavirus VP7 G and P genotypes from Iran, along with reference rotavirus isolates, underwent multiple sequence alignments. This process was conducted using BioEdit and the Clustal W method, following the methodology described by Thompson et al., in 1994 (Thompson et al., 1994). The resulting alignments were then utilized for phylogenetic analysis, employing the Maximum Likelihood (ML) method. The Kimura 2-parameter model, as implemented in MEGA 11 by Tamura et al., in 2011, was used for this analysis (Tamura et al., 2011). To determine the statistical support for the resulting trees, bootstrapping with 1000 replicates was performed. Additionally, the structural analysis of VP7 (PDB 3FMG) and VP8 (PDB 1KQR) from the Iranian sequences was carried out using the UCSF Chimera-Molecular Modeling system, following the methodology described by Pettersen et al. in 2004 (Pettersen et al., 2004).

## 3 Results

### 3.1 Positive rate of rotavirus in Iranian children between 1986 and 2023

The collective positive rate of rotavirus in 16,136 children under five years of age with acute gastroenteritis from 41 studies conducted between 1986 and 2023 was estimated to be 37.4 % (95 % CI: 32.42 %- 42.29 %). The range of positive rates varied from 10.88 % to 70.2 %. All confirmed positive cases (*n* = 6331) were verified as rotavirus positive using EIA or RT-PCR, with fewer cases confirmed using PAGE, PHT, LAT, or ICA, EM (Table 1).

**Table 1:** Characteristics of epidemiological rotavirus studies conducted in Iran during 1986–2023.

Setting	No. of AGE cases	No. of positive cases (%)	Age group	Laboratory method**^#^**	Study period	Reference
Tehran	915	228 (25)	0–5 years	PHT or LAT	1986–1987	Safieh Amini, 1990
Tehran	704	108 (15.3)	0–5 years	EIA	1993–1994	Shahrazad Modarres, 1995
Zahedan	171	50 (29.2)	0–6 years	PAGE	1993	Abdolvahhab Moradi, 2001
Ahvaz	63	23 (36.5)	1 month-2 years	PAGE	2001–2002	Samarbafzadeh et al., 2005
Shahrekord	259	91 (35)	0–5 years	EIA, EM, PAGE, RT-PCR	2001–2002	Khalili et al., 2004
Tehran	704	108 (15.3)	0–5 years	EIA	2004	Zarnani et al., 2004
Esfahan	185	57 (30.8)	0–5 years	EIA	2003–2004	Kazemi et al., 2006
Tehran	374	92 (24.5)	6 months-5 years	EIA	2003–2004	Farahtaj et al., 2007
Tehran	1250	404 (32.3)	0–5 years	EIA	2002–2005	MODARES et al., 2008
Mazandaran	400	248 (62)	1 month->10 years	EIA	2005–2006	Hamkar et al., 2010
Jahrom	102	69 (67.6)	0–5 years	EIA	2006–2007	EMAM et al., 2008
Tehran	260	91 (35)	0–5 years	EIA	2006–2007	Mohammad Kargar, 2007
Tabriz	227	111 (49)	0–5 years	EIA	2006–2007	Eesteghamati et al., 2009
Mashhad	269	133 (49.4)	0–5 years	EIA	2006–2007	Eesteghamati et al., 2009
Bandar Abbas	627	397 (63.3)	0–5 years	EIA	2006–2007	Eesteghamati et al., 2009
Tehran	250	110 (44)	0–5 years	EIA	2006–2007	Eesteghamati et al., 2009
Shiraz	825	547 (66.3)	0–5 years	EIA	2006–2007	Eesteghamati et al., 2009
Mashhad	156	45 (28.8)	0–6 years	LAT	2006–2007	Sadeghian et al., 2010
Tehran	700	131 (19)	1 month-5 years	PAGE	2004–2008	MODARRES et al., 2011
Shiraz	138	48 (34.78)	0–5 years	EIA	2007–2008	Kargar et al., 2012b
Marvdasht	141	40 (28.37)	0–5 years	EIA	2007–2008	Kargar et al., 2012
Tabriz	511	248 (55.6)	0–3 years	EIA	2007–2009	Ghorashi et al., 2011
Tehran	94	40 (42.55)	6 months-5 years	EM	2009	Ataei Pirkooh and Shahrabadi, 2007
Ahvaz	180	59 (32.7)	0–5 years	EIA	2008–2009	Mozhgani et al., 2012
Shiraz	827	347 (42)	0–12 years	EIA	2008–2010	Motamedifar et al., 2013
Borazjan,	375	91 (24.3)	0–7 years	EIA	2008–2010	Najafi et al., 2013
Borazjan	316	88 (27.85)	0–5 years	EIA	2009–2010	Kargar et al., 2011
Jahrom	163	75 (46.01)	0–5 years	EIA	2009–2010	Kargar and Akbarizadeh, 2012
Tehran	80	39 (48.8)	2 months-9 years	ICA	2009–2011	Shokrollahi et al., 2014
Shiraz, Tehran, Bandar Abbas, Tabriz, Mashhad	2988	1658 (55.48)	0–5 years	EIA	2010–2011	Jadali et al., 2012
Yasuj	184	52 (28.26)	0–5 years	EIA	2010–2011	Kargar et al., 2014
Zabol	82	57 (70.2)	0–1 year	ICA	2013	Sharifi-Rad et al., 2015
Ahvaz	200	100 (50)	0–5 years	ELISA	2011–2012	Azaran et al., 2016
Tehran	170	49 (28.8)	0–5 years	RT-PCR	2013–2014	Nasab et al., 2016
Ahvaz	100	38 (38)	0–5 years	LAT	2015–2016	Azaran et al., 2018
Gorgan	349	46 (13.18)	0–5 years	RT-PCR	2016–2017	Lorestani et al., 2019
Qom	130	22 (16.9)	0–15 years	RT-PCR	2017–2019	Shams et al., 2020
Tehran	361	110 (29.3)	0–5 years	RT-PCR	2015–2017	Motamedi-Rad et al., 2020
Tehran	200	48 (24)	0–5 years	RT-PCR	2021–2022	Kachooei et al., 2023
Mashahd	106	33 (31.3)	0–5 years	RT-PCR	2020–2022	Ketabi et al., 2023
Total*	16,136	6331 (37.4 % [95 % CI: 32.42 %- 42.29 %])			–	–

### 3.2 Distribution of circulating rotavirus strains in Iranian children between 2001 and 2023

#### 3.2.1 Overall genotype distribution

During the 20-year study period (2001–2023), genotype analysis of 964 G typed samples revealed that G1 was the most prevalent, accounting for 41.6 % (*n* = 401) of all G typed rotavirus strains. The prevalence of other G typed samples, in a descending order, was as following: G4 (12.1 %, *n* = 117), G2 (9.6 %, *n* = 93), G9 (5.5 %, *n* = 53), G3 (3.9 %, *n* = 38), G8 (2.7 %, *n* = 26), G12 (0.9 %, *n* = 9). The mixed G types (G1/G2, G1/G9, G1/G2/G4, G2/G9, G1/G8) accounted for 5.4 % (*n* = 52), while 18.2 % (*n* = 175) of samples were not typed. Similarly, genotype analysis of 672 P typed samples indicated that P[8] was the most prevalent accounting for 75 % (*n* = 505) of all P typed rotavirus strains. The prevalence of other P typed samples, in a descending order, was as following: P[4] (11.6 %, *n* = 78), P[6] (1.3 %, *n* = 9), P[9] (0.15 %, *n* = 1). The mixed P types accounted for 0.15 % (*n* = 1), while 11.6 % (*n* = 78) of samples were not typed. The G–P genotype combination was determined for 460 of the rotavirus positive samples studied between 2001 and 2023. Collectively, G1P[8] was the most frequently detected combination, accounting for 52 % (*n* = 239) of all rotavirus infections, followed by G9 G9P[8] (11.5 %, *n* = 53), G4P[8] (10.4 %, *n* = 48), G2P[4] (6.7%, *n* = 31), G3P[8] (6.7 %, *n* = 31) and G12P[8] (1.7 %, *n* = 8) (Table 2).

**Table 2:** Distribution of rotavirus G and P genotypes detected in Iran during 2001–2023.

Genotypes	2001–2012	2015–2023	All studies
P-types			
P[8]	318 (78.9 %)	187 (69.5 %)	505 (75 %)
P[4]	46 (11.4)	32 (11.9 %)	78 (11.6 %)
P[6]	0 (0 %)	9 (3.3 %)	9 (1.34 %)
P[9]	1 (0.25 %)	0 (0 %)	1 (0.15 %)
Mixed	0 (0 %)	1 (37 %)	1 (0.15 %)
NT	38 (9.4 %)	40 (14.9 %)	78 (11.6 %)
Total	403 (100 %)	269 (100 %)	672 (100 %)
G-types			
G1	332 (46 %)	69 (28.4 %)	401 (41.6 %)
G2	71 (9.8 %)	22 (9.1 %)	93 (9.6 %)
G3	4 (0.6 %)	34 (14 %)	38 (4 %)
G4	107 (14.8 %)	10 (4.1 %)	117 (12.13 %)
G8	25 (3.5)	1 (0.4 %)	26 (2.7 %)
G9	6 (0.83 %)	47 (19.3 %)	53 (5.5 %)
G12	0 (0 %)	9 (3.7 %)	9 (0.93 %)
Mixed	47 (6.5 %)	5 (2.1 %)	52 (5.4 %)
NT	129 (17.9 %)	46 (18.9 %)	175 (18.2 %)
Total	721 (100 %)	243 (100 %)	964 (100 %)
Combined genotypes^#^			
G1P[8]	175 (65.5 %)	64 (33.2 %)	239 (52 %)
G2P[4]	20 (7.5 %)	11 (5.7 %)	31 (6.7 %)
G3P[8]	1 (0.37 %)	30 (15.5 %)	31 (6.7 %)
G4P[8]	43 (16.1 %)	5 (2.6 %)	48 (10.4 %)
G9P[8]	11 (4.1 %)	42 (21.8)	53 (11.5 %)
G12P[8]	1 (0.37 %)	7 (3.6 %)	8 (1.7 %)
Uncommon			
G1P[4]	13 (4.9 %)	2(1.04 %)	15 (3.3 %)
G9P[4]	0 (0 %)	15 (7.8 %)	15 (3.3 %)
G1P[6]	0 (0 %)	1 (0.52 %)	1 (0.2 %)
G8P[8]	0 (0 %)	1 (0.52 %)	1 (0.2 %)
G2P[8]	0 (0 %)	9 (4.7 %)	9 (2 %)
G3P[6]	0 (0 %)	1 (0.52 %)	1 (0.2 %)
G4P[6]*	0 (0 %)	1 (0.52 %)	1 (0.2 %)
G9P[6]	0 (0 %)	3 (1.5 %)	3 (0.65 %)
G12P[6]	0 (0 %)	1 (0.52 %)	1 (0.2 %)
Rare			
G1P[10]	2 (0.75 %)	0 (0 %)	2 (0.43 %)
G3P[9]*	1 (0.37 %)	0 (0 %)	1 (0.2 %)
Total	267 (100 %)	193 (100 %)	460 (100 %)

⁎ Selected genotypes common in another host species (G4P[6] in pigs and G3P[9] in cats).

Furthermore, uncommon combinations of rotavirus strains were identified in 51 isolates, which included G1P[4] (3.3 %, *n* = 15), G9P[4] (3.3 %, *n* = 15), G2P[8] (1.9 %, *n* = 9), G9P[6] (0.65 %, *n* = 3), G1P[10] (0.4 %, *n* = 2), G1P[6] (0.2 %, *n* = 1), G8P[8] (0.2 %, *n* = 1), G3P[6] (0.2 %, *n* = 1), G4P[6] (0.2 %, *n* = 1), G12P[6] (0.2 %, *n* = 1), and G3P[9] (0.2 %, *n* = 1) strains. Additionally, the rare combinations G1P[10] and G3P[9] were also found in less than 0.5 % of the rotavirus strains (Table 2).

#### 3.2.2 Genotype distribution in two times intervals 2001–2012 and 2015–2023

Between 2001 and 2012, the most prevalent G genotypes were G1 (46 %, *n* = 332) and G4 (15 %, *n* = 107), followed by G2, G8, G3, and G9. However, from 2015 to 2023, the predominant G genotypes were G1 (28.4 %, *n* = 69), G9 (19.34 %, *n* = 47), and G3 (14 %), followed by G2, G4, G12, and G8. Similarly, the predominant P genotype was P[8] in both time intervals, with a prevalence of 78.9 % (*n* = 318) from 2001 to 2012 and 69.5 % (*n* = 187) from 2015 to 2023. The P[4], P[6], and P[9] genotypes followed as less common. The analysis of G-P combinations revealed that G1P[8] and G4P[8] were the dominant strains from 2001 to 2023, accounting for 65.5 % and 16.1 % of all strains, respectively. In the period from 2015 to 2023, the most prevalent genotypes were G1P[8] (33 %, *n* = 64), G9P[8] (21.7 %, *n* = 42) and G3P[8] (15.5 %, *n* = 30) followed by G9P[4] as uncommon emerging genotype. It is worth noting that although G1 and P[8] genotypes and/or G1P[8] combination continued to be predominant in both time intervals, their frequency of detection showed a decreasing trend during 2015–2023 (Table 2).

### 3.3 Phylogenetic analysis of rotavirus VP7 and VP4/VP8 genes

**Fig. 1:**
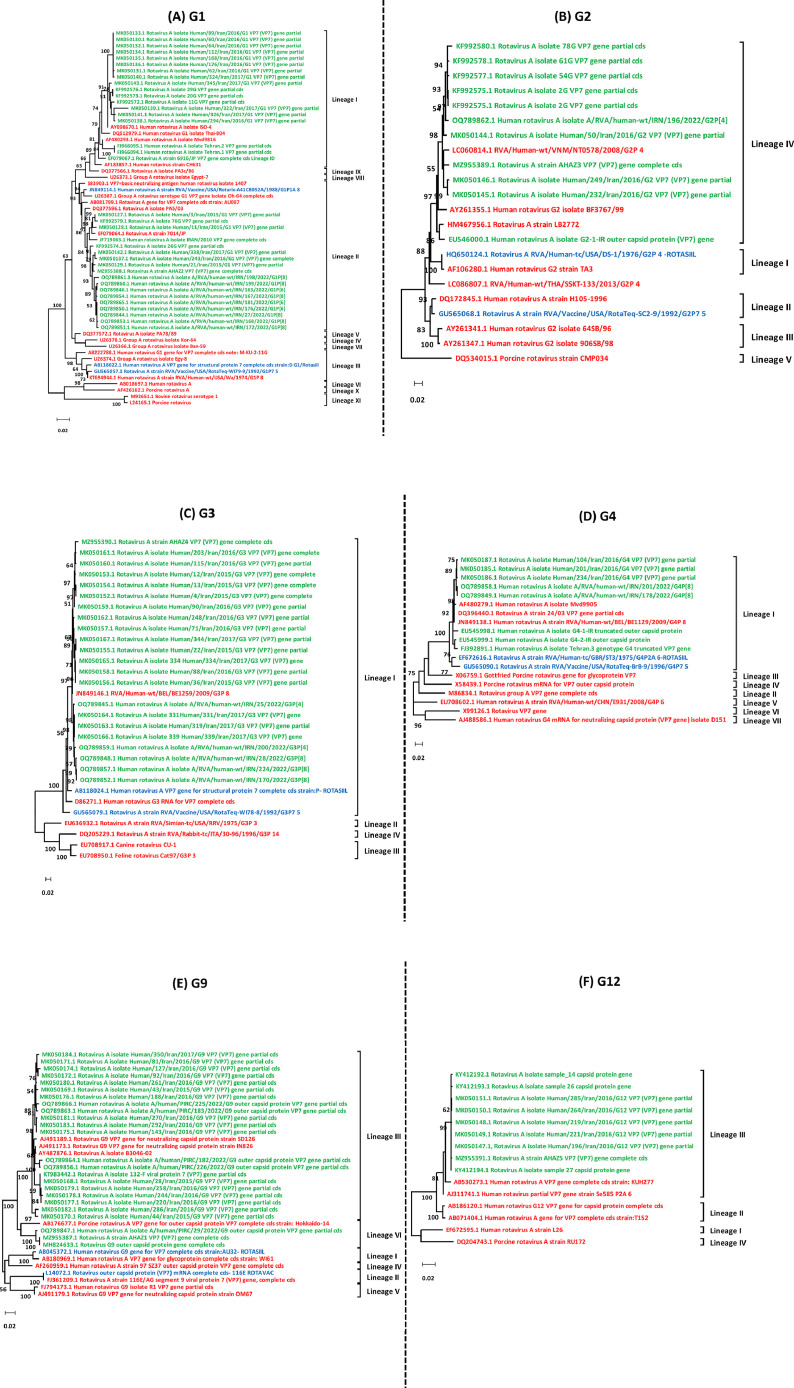

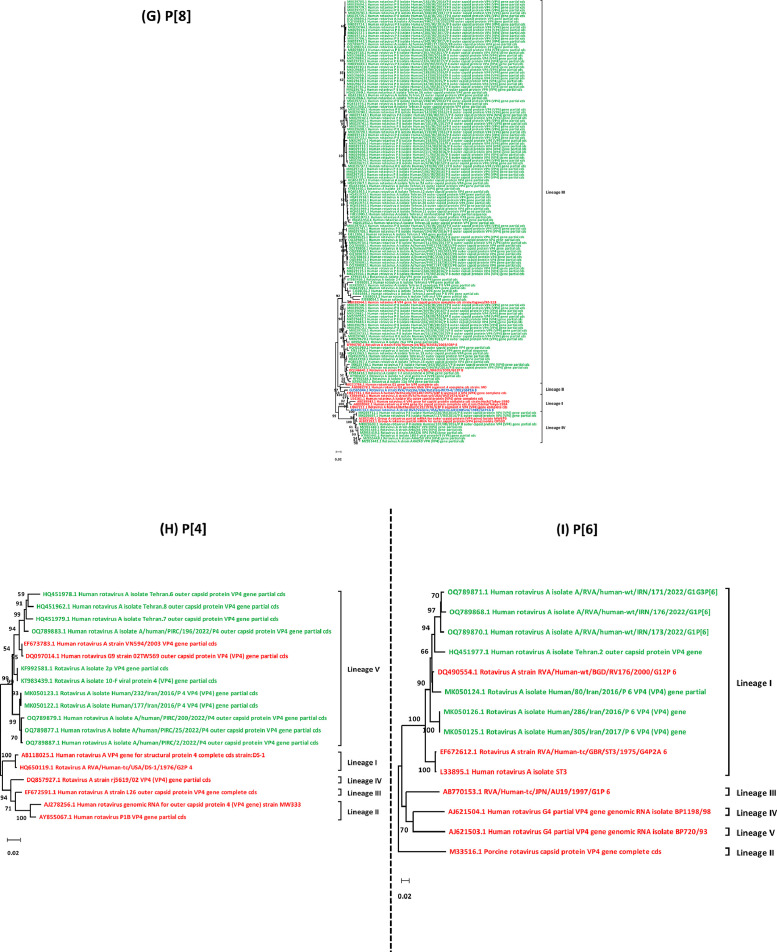
**Phylogenetic analysis based on the nucleotide sequence of the VP7 (G1-G4, G9, and G12) and VP4/VP8 genes (P[8], P[4], and P[6]**. The phylogenetic trees were constructed based on nucleotide sequence of VP7 G1 **(A),** G2 **(B),** G3 **(C),** G4 **(D),** G9 **(E),** G12 **(F),** and VP4/VP8 P[8] **(G)**, P[4] **(H)**, P[6] **(I)**. The trees were constructed through the maximum likelihood method (MLM), using the Kimura 2-parameter model. Trees were statistically supported by bootstrapping with 1000 replicates. Only bootstrap values greater than 50 % are presented. The scale bar represents 0.1 (G12, P[4]), 0.05 (G3), 0.02 (G1, G2, G4, G9, P[8], P[6]) genetic distance. The sequences of rotavirus strains identified in this study are indicated by the strain names with green squares. The reference strains are indicated by the strain names.

The phylogenetic trees were constructed using the VP7 nucleotide sequences of 110 rotavirus strains obtained from Iran (Fig. 1A-F). These strains consisted of 36 G1, 11 G2, 22 G3, 8 G4, 24 G9, and 9 G12. The VP7 sequences of G1 and G9 strains identified in Iran were categorized into two lineages: I (18 strains), II (18 strains), and two lineages III (21 strains), VI (3 strains), respectively (Fig. 1A and 1E). These lineages were statistically supported by bootstrap values ranging from 65 % to 100 %. Upon closer examination of the G1 and G9 phylogenetic trees, it was confirmed that the G1 strains identified in Iran were more closely related to the VP7 of G1 of Roarix (G1- lineage II) than to RotaTeq and ROTASIIL strains (G1- lineage III). The G9 strains identified in Iran, belonging to lineages III and VI, were relatively distantly related to VP7 of the G9 of ROTASIIL (G9- lineage I) and ROTAVAC (G9- lineage II). Additionally, the VP7 sequences of G2, G3, G4, and G12 strains identified in Iran clustered into single lineages IV, I, I, and III, respectively (Fig. 1B-D and 1F). These lineages were statistically supported by bootstrap values ranging from 99 % to 100 %. It is worth noting that the G2-lineages in both RotaTeq (G2- lineage II) and ROTASIIL (G2- lineage I) vaccines were different from the lineages of circulating G2 strains identified in Iran (G2- lineage IV), as indicated by the G2 phylogenetic analysis. However, the G3 and G4 strains formed a cluster together with the RotaTeq and ROTASIIL strains, indicating their close phylogenetic relatedness within G3 and G4- lineage I.

Similarly, the phylogenetic trees were constructed using the VP4/VP8 nucleotide sequences of 159 rotavirus strains obtained from Iran (Fig. 1G-I). These strains consisted of 141 P[8], 11 P[4], and 7 P[6]. The VP4/VP8 sequences of P[8] strains identified in Iran were categorized into two lineages: III (132 strains) and IV (9 strains) (Fig. 1G). These lineages were statistically supported by 92 % to 94 % bootstrap values. Iranian P[8]- lineage III rotavirus strains were more phylogenetically similar to P[8]- lineage II of RotaTeq than to P[8]- lineage I of Rotarix. It is also shown that P[8]- lineage IV was rather distantly phylogenetically related to RotaTeq and Rotarix. Furthermore, the VP4/VP8 sequences of P[4] and P[6] strains identified in Iran clustered to single lineages V and I, respectively (Fig. 1H and 1I). These lineages were statistically supported by 99 % to 100 % bootstrap values.

### 3.4 Comparative analysis of VP7 antigenic epitopes between Iran and vaccine strains

**Fig. 2:**
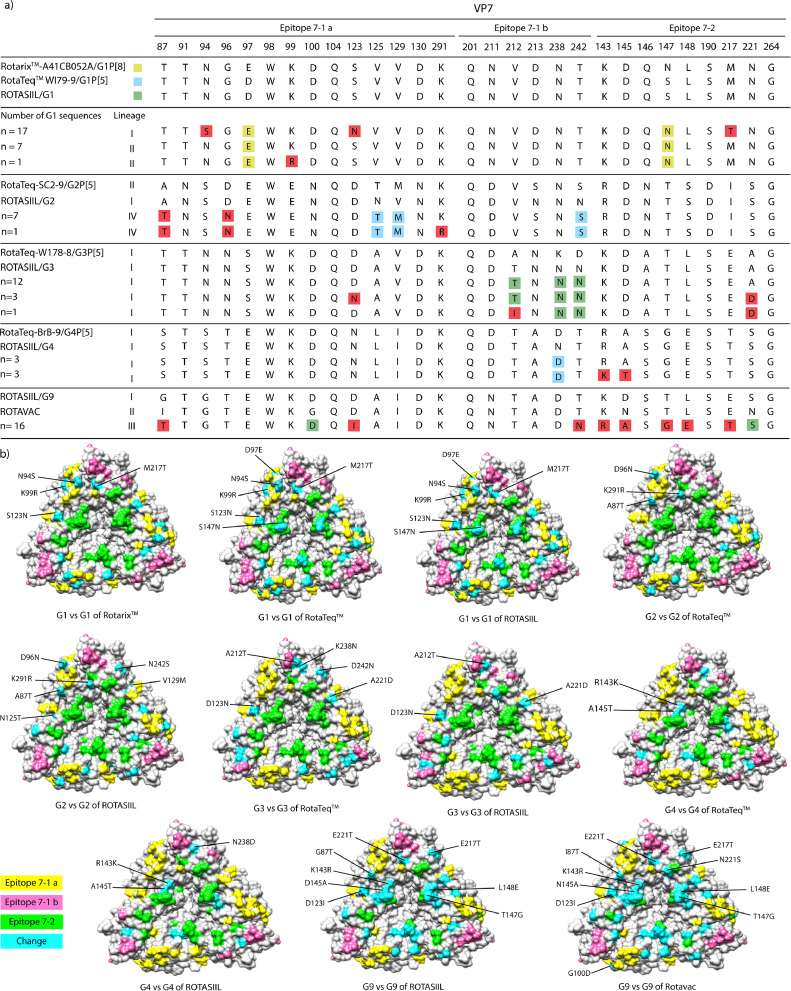
**Alignment of the antigenic epitopes in VP7 between Rotarix, RotaTeq, ROTAVAC, and ROTASIIL vaccines and strains circulating in Iran**. Alignment of the antigenic epitopes in VP7 of the rotavirus strains circulating in Iran with Rotarix, RotaTeq, ROTASIIL, and ROTAVAC. Residues that differ from Rotarix, RotaTeq, ROTASIIL, and ROTAVAC are highlighted in red; residues similar to Rotarix are shaded in pea green, those similar to Rotateq in blue, and those similar to ROTASIIL in teal **(A)**. Surface representation cartoon of the VP7 monomer and trimer (PDB 3FMG). Antigenic epitopes are colored yellow (7–1a), pink (7–1b), and green (7–2). Substitutions relevant to the vaccine strains are depicted in blue **(B)**.

The VP7 trimer consists of three antigenic epitopes, 7–1a, 7–1b, and 7–2. A comparison of the VP7 antigenic epitopes in G1-lineages I and II strains found in Iran with G1-lineages II and III in Rotarix and RotaTeq/ROTASIIL vaccines revealed three amino acid substitutions of N94S, S123N, and M217T when compared to G1 of Rotarix. Additionally, five substitutions of N94S, S123N, M217T, D97E, and S147N were identified in comparison with G1 of RotaTeq, and seven substitutions of N94S, S123N, M217T, D97E, and S147N in comparison with G1 of ROTASIIL (Fig. 2). It was observed that G1- lineage I had more amino acid differences (positions 94, 97, 123, 147, and 217) with the vaccine strains compared to G1- lineage II (positions 97 and 147). When comparing the VP7 antigenic epitopes of G2-lineage IV strains with G2-lineages II and I strains of RotaTeq and ROTASIIL, four amino acid substitutions (A87T, D96N, S213D, and S242N) were identified in 7–1a and 7–1b epitopes (Fig. 2). Furthermore, the comparison of VP7 epitopes in G3-lineage I strains found in Iran with G3-lineage I RotaTeq and ROTASIIL strains showed three amino acid substitutions (A212T, K238N, D242N) located exclusively within the 7–1b epitope (Fig. 2). Lastly, the VP7 antigenic epitopes of G4 Lineage I strains identified in Iran exhibited five residue substitutions in comparison with the antigenic epitopes of G4-lineage I strains of RotaTeq and ROTASIIL (T87S, D130E, R143K, A145T, and D211N), present in all three antigenic epitopes (Fig. 2). An analysis of the VP7 antigenic epitopes of the G9- lineages III/IV strains found in Iran, in comparison with the G9- lineages I and II strains of ROTASIIL and ROTAVAC, respectively, revealed that the G9- lineage III/IV strains in Iran exhibited three amino acid substitutions (A212T, K238N, D242N) when compared to the G3 strain of ROTASIIL and ROTAVAC vaccines. These substitutions were specifically located within the 7–1b epitope (Fig. 2).

### 3.5 Comparative analysis of VP4/VP8 antigenic epitopes between Iran and vaccine strains

**Fig. 3:**
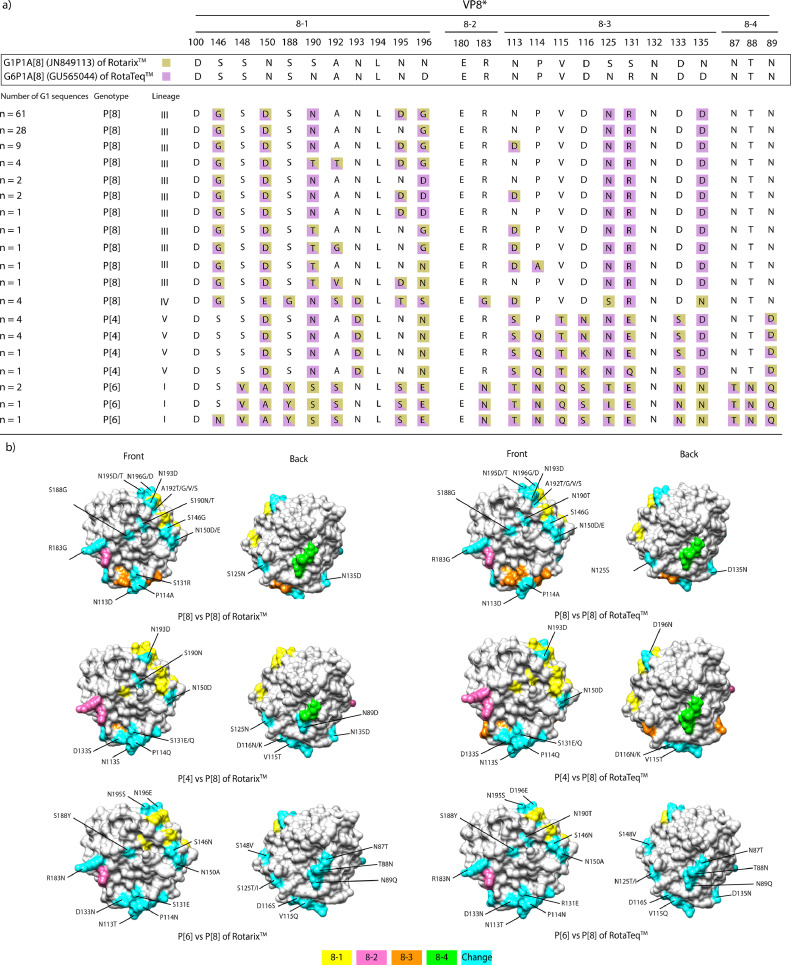
Alignment of the antigenic epitopes in VP4/VP8* between Rotarix/RotaTeq vaccines and strains circulating in Iran. Alignment of the antigenic epitopes in VP8 between Rotarix and RotaTeq, and strains circulating in Iran. Amino acid residues shaded in pink and green differ from Rotarix and RotaTeq, respectively; amino acid residues highlighted in pink/green differ from both Rotarix and RotaTeq **(A)**. The VP8 core is represented on the surface (PDB KQR). Antigenic epitopes are highlighted in yellow (8‐1), pink (8‐2), orange (8‐3), and green (8‐4). Surface-exposed residues that differ between P[8] strains and vaccine strains are shown in light blue **(B)**.

The VP8* head contains four surface-exposed antigenic epitopes (8–1 to 8–4) that are expected to consist of 25 amino acids. A comparison of the VP8 antigenic epitopes between P[8]-lineages III and IV, identified in Iran, and the P[8]-lineages I/II Rotarix/RotaTeq vaccines revealed that there were 8 to 9 substitutions of P[8]-lineage III amino acids in Rotarix and 4 to 6 substitutions in RotaTeq (Fig 3). Additionally, P[8]-lineage IV reported 11 differences per strain (Fig. 3). Furthermore, the VP8* antigenic epitopes of the P[4]-lineage V strains identified in Iran showed 11–12 and 9–10 residue substitutions when compared to Rotarix and RotaTeq strains, respectively (Fig. 3). The P[6]-lineage I strains identified in Iran exhibited the highest divergence, with 17–20 residue substitutions, from the P[8] antigenic epitopes of the vaccine strains, which were dispersed across all antigenic epitopes 8–1, 8–2, 8–3, and 8–4.

## 4 Discussion

The current study presents a comprehensive analysis of rotavirus infections in children under five years of age who were hospitalized with acute gastroenteritis in Iran from 1986 to 2023 (before rotavirus vaccination program) with a particular emphasis on the distribution patterns of circulating genotypes/sublineages as well as genetic/antigenic and structural differences of specially VP7 and VP8 antigens compared to that of the rotavirus vaccine strains (Rotarix, RotaTeq, ROTAVAC, and ROTASIIL) to provide insights into the optimal rotavirus vaccination policy for Iran and region. The overall data reveals that approximately 38.1 % of the tested samples were positive for rotavirus, which aligns with findings from countries that have not yet implemented rotavirus vaccination in their national immunization programs (Aliabadi et al., 2019). When examining the distribution of rotavirus genotypes in Iran, it is evident that the G1 and P[8] genotypes, as well as the G1P[8] combination, are the most prevalent genotypes/combinations. Interestingly, between 2015 and 2023, there has been a decline in the prevalence of G1P[8] accompanied by an increase in G3P[8] and the emergence of G9P[8]. Additionally, the emergence of the uncommon genotype G9P[4] should not be overlooked. Previous studies have shown that the dominant strains of rotavirus naturally fluctuate over a period ranging from 3 to 11 years (Arista et al., 2006; Bányai et al., 2009; Cilla et al., 2010; Pitzer et al., 2011). In this regard, a mathematical model was developed to analyze the dynamics of rotavirus strains, focusing on the interaction between five common genotypes (G1-G4 and G9) (Pitzer et al., 2011). Accordingly, when G1 strains are prevalent, infants have a lower susceptibility to a second infection with a G1 strain compared to an infection with a heterotypic (rare) strain. As a result, the rare genotypes have a fitness advantage because they are more capable of infecting individuals with homotypic immunity to G1. The stronger the homotypic immunity relative to heterotypic immunity, the faster cycling is expected to occur, as the rare genotypes will gain a fitness advantage at the population level more quickly. It has been suggested that cross immunity can lead to cyclical patterns in incidence and distribution of strains (Pitzer et al., 2011). In Iran, G1 and P[8] genotypes and/or G1P[8] combination were prevalent in children during two time intervals: 2001–2012 and 2015–2023, with a decreasing trend from 2012 to 2023. To support these annual fluctuations, G3 and G9 were reported to be predominant or co-circulate with G1 (Ghanaiee et al., 2023; Kachooei et al., 2023; Motamedi-Rad et al., 2020). Therefore, it is suggested that a stronger homotypic immunity against G1P[8] will likely lead to an increase in the relative prevalence of non-G1 strains (G9P[8], G3P[8], and G9P[4]). A similar study in South Africa reported that G2 strains were dominant or co-dominant with G1 every 3 to 4 years (Page and Steele, 2004). Furthermore, the observed variability in the dominant periods of oscillations may be due to the inherent stochasticity resulting from occasional fadeouts of strains, the short length of available time-series data compared to observed multiannual periodicities, and potential differences in transmission dynamics among countries. However, more comprehensive multiyear data is needed to address fluctuations in genotype distribution.

Concerning the selection of the most proper rotavirus vaccine for national vaccination program in Iran, various scenarios can be anticipated depending on the type of vaccines used and the circulating rotavirus genotypes/ lineages, regardless of P genotype approach. In the first scenario, where Rotarix vaccine is chosen for vaccination, the phylogenetic analyses of the VP7 sequence of G1 genotype reveal that all G1 strains identified in Iran belong to G1-lineages I and II. These lineages exhibit the closest phylogenetic relationship with the G1-lineage II strain of Rotarix, as compared to RotaTeq and ROTASIIL vaccines strains. Furthermore, a closer examination of the three immunodominant region recognized in the VP7 and its antigenic epitopes reveals that all 29 amino acid residues of these epitopes are completely conserved among all G1- lineage II identified in Iran and Rotarix strain. In contrast, G1- lineage I shows three amino acid substitutions (N94S, S123N, and M217T) when compared with Rotarix strain. Additionally, there are 12–18 residues dispersed in the 7–1a, 7–1b, and 7–2 epitopes that exhibit relative differences among heterotypic strains (G2-G4, G9, and G12) identified in Iran and Rotarix. The available data indicates that the use of Rotarix vaccination provides strong protection against homotypic strains G1 and partial protection against other circulating heterotypic and new strains. It is expected that the heterotypic strain will take a few years (2 to 3 years) to become established in the population, causing a normal-sized epidemic and circulating alongside other genotypes. However, despite an increase in infections caused by non-G1 strains, the overall incidence is significantly reduced.

In the case of using the ROTAVAC vaccine (the second scenario), the phylogenetic analysis of G9 genotype in Iran shows that the identified G9 strains are distantly related to the G9 of ROTAVAC (G9- lineage II), which belongs to G9- lineages III and IV. Additionally, there are seven amino acid substitutions in the antigenic epitopes of G9- lineages III and IV in Iran, which differ from the ROTAVAC strain. These substitutions are mainly found in the neutralization epitopes of the VP7 protein, potentially affecting the efficacy of ROTAVAC. Since the ROTAVAC strain contains VP4 with genotype P[11], which has not been detected in Iran, the protective approach of this vaccine in Iran would primarily rely on the G9 genotype (except for VP6 protein, which is partially effective for all live attenuated rotavirus vaccines). However, the extent of cross-protection against non-G9 genotypes circulating in Iran remains uncertain. Hence, considering the disparity in G9 lineages between the vaccine and the circulating strains in Iran, there is apprehension regarding its ability to offer adequate protection against the circulating G9 strain. To support this notion, studies have shown that antisera raised against G9 lineage II or III strains exhibit a lower ability to neutralize other G9 lineages rotaviruses (Hoshino et al., 2004). Conversely, antibodies against G9 lineage I strains were found to neutralize not only lineage I strains but also lineage II and III strains at high levels (Hoshino et al., 2004). Therefore, it is crucial to develop a G9-based vaccine that is based on the strain with the broadest reactivity, namely G9- lineage I. Considering the genetic and antigenic characterization of G9 strains in Iran and that of the ROTAVAC, it is important to note that the emergence of G9 lineages different from the ROTAVAC strain raises concerns about the efficacy of the ROTAVAC vaccine. Indeed, this raises doubts about the vaccine's ability to provide strong homotypic protection against G9 lineages and heterotypic protection against non-G9 strains. While it is predicted to primarily protect against G9, G1 remains the dominant genotype and continues to circulate alongside other non-G9 genotypes.

In the context of vaccination, the third scenario involves the use of RotaTeq and ROTASIIL (polyvalent) vaccines. Through phylogenetic analysis, it has been determined that the VP7 gene G1- lineages I and II strains found in Iran are relatively distant from the VP7 sequence of G1- lineage III strains in RotaTeq and ROTASIIL vaccines. This difference is attributed to five amino acid substitutions (N94S, S123N, M217T, D97E, and S147N) in antigenic epitopes, with the latter two substitutions (D97E and S147N) being particularly important for virus neutralization (Aoki et al., 2009). Furthermore, there is significant diversity in the VP7 sequence between the G2- lineage IV strains identified in Iran and the G2- lineage II of RotaTeq/G2- lineage I of ROTASIIL vaccines. This diversity is characterized by amino acid substitutions (A87T, D96N, N125T, V129M, S/N213D, S242N) in antigenic epitopes, which may be associated with the re-emergence of G2 strains (Gomara et al., 2001; Zao et al., 1999) and potentially lead to a decrease in vaccine efficacy against G2 strains. On the other hand, the lineage I of G3 and G4 strains identified in Iran are closely related to the G3/G4 strains of RotaTeq, belonging to the same lineage. However, there are two amino acid substitutions (A212T and D242N) in the 7–1b region identified in Iran when compared to RotaTeq. Additionally, the G3 strains identified in Iran also exhibit the K238N amino acid substitution (compared to both RotaTeq and ROTASIIL vaccines), which creates a potential N-linked glycosylation site strains (Morozova et al., 2015; Mouna et al., 2013; Zeller et al., 2012) This substitution may also impact the neutralization activity against homotypic rotavirus strains (Ciarlet et al., 1997, 1994). Similarly, the alignment of amino acid residues in the VP7 antigenic epitopes revealed that the RotaTeq G4 strain had five substitutions at sites 87, 130, 211, 143, and 145. On the other hand, the ROTASIIL G4 strain showed two substitutions at sites 238 and 145. These findings suggest that both the RotaTeq and ROTASIIL vaccines can offer equally strong protection against all G1-G4 strains. However, it is anticipated that the emergence of a new strain may result in the extinction of less-transmissible genotypes (such as G2-G4 strains), leading to the continued predominance of G1. The new strain is anticipated to circulate along with G1 and may also prolong the period of oscillations for up to 8 years. Notably, the immunogenic epitopes of ROTASIIL strains were found to be slightly more closely related to the circulating G1-G4 strains identified in Iran compared to RotaTeq. Additionally, the ROTASIIL vaccine's G9-lineage I strain component, which differed from the G9-lineage III strain identified in Iran by two substitutions at sites 87 and 242, has been shown to neutralize not only lineage I strains but also lineage II and III strains at high titers (Hoshino et al., 2004) (Hoshino et al., 2004). Considering these factors, it is reasonable to suggest that vaccination with the ROTASIIL vaccine would be more effective in Iran compared to RotaTeq.

The role of VP8* in rotavirus binding to cells has been reevaluated following the discovery of HBGAs as potential rotavirus receptors (Hu et al., 2012; Huang et al., 2012; Liu et al., 2012). This has led to proposition of novel approaches by using VP8* in the improvement of the already available rotavirus vaccines, as well as development of VP8*-based subunit vaccines. Indeed, the P[4], P[6], and P[8] genotypes of VP4/VP8 account for the majority (over 95 %) of detected rotavirus strains (Doro et al., 2014), with P[8] alone representing 90 % of all rotavirus infections. Of note, the P[8] genotype specifically recognizes the Lewis b and H1-type antigens of HBGAs (secretor status), which can impact susceptibility to rotavirus gastroenteritis. Hence, based on the P genotype approach, a fourth scenario is proposed where the VP8 protein plays a crucial role in the protection provided by available vaccines. This protein possesses neutralizing and antigenic properties that are of potential importance in vaccine implications (Zeller et al., 2012). In this regard, both Rotarix and RotaTeq vaccines are shown to offer equal protection against human rotaviruses and provide cross-protection between different G-genotypes, which typically have the same P genotype (P[8] being the most prevalent worldwide. This may explain why both vaccines provide similar levels of protection. In Iran, the distribution of P genotypes indicates that the most prevalent rotavirus genotype is P[8]. Given the secretor and Lewis-positive status of most individuals in Europe, North American countries, and other Caucasians, such as Iranians, it is not surprising that the majority of rotaviruses detected in Iran belong to the P[8] genotypes. (Carlsson et al., 2009; Farahmand et al., 2021a, 2021b; Le Pendu, 2004). This is further supported by the fact that Rotarix and RotaTeq vaccines, which are commonly used to combat rotavirus gastroenteritis, primarily target the P[8] genotype. Hence, vaccination with these two vaccines is expected to be effective in Iran, while this might not be relevant for ROTASIIl and ROTAVAC vaccines. However, it is important to note that the P[8] lineages identified in Iran differ from the vaccine strains. Indeed, upon closer examination of the phylogenetic tree and antigenic epitopes of P[8] sequences in Iran, it was observed that most Iranian strains belong to P[8]-lineage III, while Rotarix and RotaTeq vaccines cluster in P[8]-lineages I and II, respectively. Additionally, the antigenic epitopes of P[8] lineages III and IV circulating in Iran differ from those of the vaccine strains. Although similar patterns of P[8]-lineage III have been documented in other countries, the variations in the VP4 genes of circulating rotavirus and vaccine strains do not seem to affect the efficacy of the vaccines.(Ruiz-Palacios et al., 2006; Vesikari et al., 2007). In fact, when a child is infected with a P[8] strain, the variability in the VP7 genes can potentially weaken the immunity provided by vaccines. However, the VP4 portion of the vaccine strains may be able to compensate for the reduced neutralizing capacity of the vaccine-induced antibodies. On the other hand, if the infecting strain's VP4 genotype is non-P[8], the effectiveness of the vaccines relies on the VP7 portion. As a result, the monovalent rotavirus vaccine Rotarix is believed to be effective against non-G1P[8] rotavirus strains. This cross defense is likely due to the presence of conserved immunogenic regions in both VP7 and VP4 genotypes, as well as non-structural or internal proteins.

The P genotype approach involves the P[11] rotavirus (ROTAVAC strain) which commonly infects neonates. Neonates recognize a type 2 HBGA precursor (Galß1–4GlcNAc) (Liu et al., 2013; Ramani et al., 2013), as a host ligand or receptor. The age at which Galß1–4GlcNAc turns on could be crucial in determining the susceptibility of P[11] in neonates/infants compared to older children. The binding of VP8* to Galß1–4GlcNAc before weeks 13 differs significantly from that of weeks 26 and 52 (Liu et al., 2013), indicating an age range for the synthesis of this precursor to turn off or for other glycotransferases to turn on. This difference could also be due to further modification of Galß1–4GlcNAc by variable glycotransferases, which may impact its interaction with P[11] rotaviruses by blocking or masking the Galß1–4GlcNAc residues. Therefore, it raises the question of whether vaccine uptake and response may vary depending on the age of vaccination and the VP4 genotype of the vaccine virus and glycan profile of the vaccinated infant. In the case of Iran, one concern regarding the use of the ROTAVAC vaccine is its effectiveness and the ability of the vaccine strain to multiply in the intestines of the Iranian population. This ability may vary among different communities due to differences in the regulation of HBGAs during development. This highlights the importance of conducting vaccine efficacy studies in various settings.

Taken together, the present review article might offer valuable information, concepts and scenarios for choosing the most effective and suitable vaccine for protection against circulating rotavirus strains in Iran and similar regions. Based on our analyses, the Rotarix vaccine, or monovalent vaccines containing the G1P[8] component, appear to be a priority for areas with a rotavirus genotypic pattern and genetic background similar to that of the Iranian population. This preference is based on positive factors such as the continued predominance of the G1 and P[8] strains, the ability to bind to secretors, and the ease of technology transfer for single or monovalent strains. While similar considerations can be made for RotaTeq and ROTASIIL vaccines, their reassortment-based technological framework does not make them a priority. However, the ROTASIIL vaccine, despite the VP4 arm (P [5]) not being considered a suitable protection option, has previously demonstrated the ability to neutralize not only G9-lineage I strains but also other G9- lineages at high titers. Therefore, it could be expected that vaccination with ROTASIIL would be more effective in Iran and similar regions compared to RotaTeq. However, in case of the ROTAVAC vaccine, considering the rotavirus genotypic pattern identified in Iran and the characteristics of ROTAVAC, it may not provide as much protection as the other vaccines.

## CRediT authorship contribution statement

**Somayeh Jalilvand:** Writing – original draft, Visualization, Resources, Formal analysis. **Tayebeh Latifi:** Software, Formal analysis. **Atefeh Kachooei:** Validation, Software, Data curation. **Mahtab Mirhoseinian:** Validation, Software, Data curation. **Somayeh-Sadat Hoseini-Fakhr:** Validation, Software, Data curation. **Farzane Behnezhad:** Validation, Software, Data curation. **Farzin Roohvand:** Writing – review & editing. **Zabihollah Shoja:** Writing – review & editing, Writing – original draft, Visualization, Validation, Supervision, Software, Resources, Project administration, Methodology, Funding acquisition, Formal analysis, Data curation, Conceptualization.

## Declaration of competing interest

The authors declare that they have no known competing financial interests or personal relationships that could have appeared to influence the work reported in this paper.

## Data Availability

Data will be made available on request.
